# Supplementary material for: Engineered helicase replaces thermocycler in DNA amplification while retaining desired PCR characteristics
Source: Nat Commun. 2022 Oct 23;13:6312. doi: 10.1038/s41467-022-34076-0 (PMC9588791; doi:10.1038/s41467-022-34076-0)
Supplement: Supplementary file 1 — Supplementary Information [file 41467_2022_34076_MOESM1_ESM.pdf]

Supplementary Information for

Engineered helicase replaces thermocycler in DNA amplification while retaining desired PCR characteristics

Author list

Momčilo Gavrilov[1], Joshua Y.C. Yang[2], Roger S. Zou[2], Wen Ma[3], Chun-Ying Lee[4], Sonisilpa Mohapatra[1], Jimin Kang[4], Ting-Wei Liao[4], Sua Myong[4], and Taekjip Ha[1,2,4,5*]

Affiliations

[1]Department of Biophysics and Biophysical Chemistry, Johns Hopkins University School of Medicine, Baltimore, MD, USA

[2]Department of Biomedical Engineering, Johns Hopkins University School of Medicine, Baltimore, MD, USA

[3]Department of Chemistry and Biochemistry, University of California, San Diego, USA

[4]Department of Biophysics, Johns Hopkins University, Baltimore, MD, USA

[5]Howard Hughes Medical Institute, Baltimore, MD, USA

e-mail: tjha@jhu.edu

Supplementary Note 1: SHARP activity versus DTT concentration

We explored conditions for the disulfide bond formation in PcrA M6 mutant and tested how DTT affects SHARP. We found that an increased DTT concentration (>50 mM) inhibits SHARP (Supplementary Fig. 2a), because no increase in the product amount was detected. This is expected because DTT breaks disulfide bonds essential for the superhelicase activity of PcrA M6 in SHARP. For a crosslinked PcrA-X M6 with BM(PEG)2 (Supplementary Fig. 2b), we observed amplification at higher DTT concentrations. This assay demonstrated that confining a PcrA M6 to its closed conformation either with disulfide bond formation or with an external crosslinker is essential for SHARP.

Supplementary Note 2: Sequences

Spinach64 sequence used in SPRNT assay consists of 8 repeats of the sequence below:

(tagacgGCATGGGGAGACGCGACCGAAATGGTGAAGGACGGGTCCAGTGCTTCGGCACTGT
TGAGTAGAGTGTGAGCTCCGTAACTGGTCGCGTCACgtaagatgctccggttaGGGAGACGCGACC
GAAATGGTGAAGGACGGGTCCAGTGCTTCGGCACTGTTGAGTAGAGTGTGAGCTCCGTAAC
TGGTCGCGTCACtgatgtaccgttgagcaGGGAGACGCGACCGAAATGGTGAAGGACGGGTCCAGT
GCTTCGGCACTGTTGAGTAGAGTGTGAGCTCCGTAACTGGTCGCGTCACtcgctagagcatggtttG
GGAGACGCGACCGAAATGGTGAAGGACGGGTCCAGTGCTTCGGCACTGTTGAGTAGAGTG
TGAGCTCCGTAACTGGTCGCGTCACtggggcacgccgtctggGGGAGACGCGACCGAAATGGTGAA
GGACGGGTCCAGTGCTTCGGCACTGTTGAGTAGAGTGTGAGCTCCGTAACTGGTCGCGTCA
CttactgcgaccgcaataGGGAGACGCGACCGAAATGGTGAAGGACGGGTCCAGTGCTTCGGCACT
GTTGAGTAGAGTGTGAGCTCCGTAACTGGTCGCGTCACgcgcgcaaccgggtagaGGGAGACGCG
ACCGAAATGGTGAAGGACGGGTCCAGTGCTTCGGCACTGTTGAGTAGAGTGTGAGCTCCGT
AACTGGTCGCGTCACgtaactcacggcgctatGGGAGACGCGACCGAAATGGTGAAGGACGGGTCC
AGTGCTTCGGCACTGTTGAGTAGAGTGTGAGCTCCGTAACTGGTCGCGTCACGCTAGA)

Sequence highlighted in green represents random linkers between Spinach repeats. Sequence highlighted in gray and underlined is a part of Spinach. The sequence highlighted in gray generates a characteristic high electric current signal sandwiched between low current signal and is marked in gray in in Figs. 7f and 7g, and Supplementary Figs. 2c, and 2d. The marked signal is easy to visually identify and is useful for counting number of spinach repeats passing through the nanopore. By counting repeats, we determine helicase average speed from the raw signal.

Amino acid sequence for engineered PcrA M6 superhelicase
H93A, C96A, N187C, C247A, L384V, L409C. The sequence does not include any tags

MNFLSEQLLAHLNKEQQEAVRTTEGPLLIMAGAGSGKTRVLTHRIAYLMAEKHVAPWNILAITFTN
KAAREMRERVQSLLGGAAEDVWISTFASMAVRILRRDIDRIGINRNFSILDPTDQLSVMKTILKEK
NIDPKKFEPRTILGTISAAKNELLPPEQFAKRASTYYEKVVSDVYQEYQQRLLRCHSLDFDDLIMT
TIQLFDRVPDVLHYYQYKFQYIHIDEYQDTNRAQYTLVKKLAERFQNIAAVGDADQSIYRWRGADI
QNILSFERDYPNAKVILLEQNYRSTKRILQAANEVIEHNVNRKPKRLWTENPEGKPILYYEAMNEA
DEAQFVAGRIREAVERGERRYRDFAVLYRTNAQSRVMEEMLLKANIPYQIVGGVKFYDRKEIKDI
LAYLRVIANPDDDCSLLRIINVPKRGIGASTIDKLVRYAADHELSLFEALGELEMIGLGAKAAGALA
AFRSQLEQWTQLQEYVSVTELVEEVLDKSGYREMLKAERTIEAQSRLENLDEFLSVTKHFENVS
DDKSLIAFLTDLALISDLDELNGTEQAAEGDAVMLMTLHAAKGLEFPVVFLIGMEEGIFPHNRSLE
DDDEMEEERRLAYVGITRAEEELVLTSAQMRTLFGNIQMNPPSRFLNEIPAHLLETASRRQAGAS
RPAVSRPQASGAVGSWKVGDRANHRKWGIGTVVSVRGGGDDQELDIAFPSPIGIKRLLAKFAPI
EKV

Amino acid sequence for the wild type PcrA (NCBI Reference Sequence WP_033016687.1[https://www.ncbi.nlm.nih.gov/protein/WP_033016687.1] is given below

MNFLSEQLLAHLNKEQQEAVRTTEGPLLIMAGAGSGKTRVLTHRIAYLMAEKHVAPWNILAITFTN
KAAREMRERVQSLLGGAAEDVWISTFHSMCVRILRRDIDRIGINRNFSILDPTDQLSVMKTILKEK
NIDPKKFEPRTILGTISAAKNELLPPEQFAKRASTYYEKVVSDVYQEYQQRLLRNHSLDFDDLIMT
TIQLFDRVPDVLHYYQYKFQYIHIDEYQDTNRAQYTLVKKLAERFQNICAVGDADQSIYRWRGADI
QNILSFERDYPNAKVILLEQNYRSTKRILQAANEVIEHNVNRKPKRLWTENPEGKPILYYEAMNEA
DEAQFVAGRIREAVERGERRYRDFAVLYRTNAQSRVMEEMLLKANIPYQIVGGLKFYDRKEIKDI
LAYLRVIANPDDDLSLLRIINVPKRGIGASTIDKLVRYAADHELSLFEALGELEMIGLGAKAAGALAA
FRSQLEQWTQLQEYVSVTELVEEVLDKSGYREMLKAERTIEAQSRLENLDEFLSVTKHFENVSD
DKSLIAFLTDLALISDLDELNGTEQAAEGDAVMLMTLHAAKGLEFPVVFLIGMEEGIFPHNRSLED
DDEMEEERRLAYVGITRAEEELVLTSAQMRTLFGNIQMNPPSRFLNEIPAHLLETASRRQAGASR
PAVSRPQASGAVGSWKVGDRANHRKWGIGTVVSVRGGGDDQELDIAFPSPIGIKRLLAKFAPIE
KV

Plasmid sequence for engineered PcrA M6_H93A, C96A, N187C, C247A, L384V, L409C was synthetized by GenScript. Vector name: pET-11b, cloning site: NdeI/BamHI, Variant sequence includes N terminal 6xHis tag and C-terminal FLAG tag:

CATATGCATCACCATCACCATCACGGGTCCGGAATGAACTTTTTATCGGAACAGCTGCTCGC
CCATTTAAACAAAGAGCAACAAGAAGCCGTCAGGACGACGGAAGGCCCGCTGCTCATTATG
GCGGGGGCGGGAAGCGGGAAAACGCGGGTGTTGACGCACCGCATCGCCTATTTGATGGCG
GAAAAGCATGTGGCGCCGTGGAACATTTTGGCCATTACGTTTACGAACAAGGCGGCGCGCG
AAATGCGGGAACGTGTGCAGTCGCTCTTAGGTGGGGCGGCGGAAGACGTCTGGATTTCGA
CGTTCGCGTCGATGGCCGTCCGCATTTTGCGCCGCGACATTGACCGCATCGGCATCAACCG
CAATTTTTCCATCCTTGATCCGACGGACCAGCTTTCAGTCATGAAAACGATTTTAAAAGAAAA
AAACATAGACCCGAAAAAATTTGAGCCGCGGACGATTTTAGGCACGATCAGCGCGGCGAAA
AACGAGCTGTTGCCTCCGGAGCAATTCGCGAAGCGGGCCTCGACGTATTACGAAAAAGTCG

TCAGCGATGTGTATCAAGAATACCAACAGCGCCTGCTTCGCTGTCATTCGCTCGATTTTGAC
GATTTGATCATGACGACGATCCAACTGTTTGACCGCGTGCCGGATGTGCTTCACTATTACCA
ATATAAGTTTCAGTACATTCATATTGATGAGTACCAGGATACGAACCGCGCTCAATATACGCT
GGTCAAAAAGCTGGCGGAACGCTTTCAAAACATTGCCGCCGTCGGCGACGCCGACCAATCG
ATTTATCGGTGGCGCGGGGCGGACATCCAAAACATTTTGTCGTTCGAGCGCGACTATCCGA
ACGCAAAAGTCATTTTGCTTGAACAAAACTACCGCTCGACGAAGCGCATTTTGCAAGCGGCG
AACGAAGTCATCGAGCATAACGTCAACCGGAAGCCGAAACGGCTTTGGACGGAAAACCCGG
AAGGAAAGCCGATTCTTTATTATGAGGCGATGAACGAAGCGGACGAAGCGCAGTTTGTCGC
TGGACGCATCCGCGAGGCGGTGGAGCGCGGCGAACGCCGCTACCGTGATTTTGCTGTCTT
GTACCGGACGAACGCCCAGTCGCGTGTCATGGAGGAAATGTTGCTGAAAGCGAACATTCCG
TATCAAATTGTCGGCGGCGTAAAGTTCTATGACCGGAAAGAAATTAAAGACATTCTCGCCTAT
TTGCGCGTCATTGCCAATCCGGACGATGATTGCAGCTTGCTTCGCATCATTAACGTGCCAAA
ACGCGGCATTGGCGCCTCGACGATCGACAAACTCGTCCGCTATGCAGCCGATCATGAGCTG
TCCTTGTTTGAGGCGCTCGGCGAGCTAGAGATGATCGGGCTTGGCGCCAAAGCGGCCGGG
GCGCTCGCCGCGTTCCGCAGCCAGCTCGAGCAATGGACACAGCTGCAAGAATACGTCTCC
GTCACCGAACTCGTCGAAGAAGTGCTCGACAAATCGGGCTACCGCGAGATGCTCAAGGCG
GAGCGGACGATTGAAGCACAAAGCCGGCTCGAGAACTTGGATGAGTTTTTGTCGGTGACGA
AGCATTTTGAAAATGTGAGCGACGATAAATCGCTCATCGCCTTTTTAACCGACTTGGCGCTC
ATTTCCGATTTGGACGAGCTGAACGGGACGGAACAGGCCGCTGAAGGAGATGCCGTCATGT
TGATGACGTTGCATGCCGCCAAAGGGCTCGAGTTTCCGGTCGTCTTTTTGATCGGCATGGA
AGAAGGCATTTTCCCGCACAACCGCTCTCTCGAGGATGACGATGAGATGGAAGAAGAACGG
CGGCTGGCGTACGTCGGCATCACCCGCGCGGAGGAAGAACTTGTGCTGACGAGCGCGCAA
ATGCGGACGTTGTTTGGCAACATCCAAATGAACCCGCCGTCGCGCTTTTTGAATGAAATTCC
GGCGCATTTGCTTGAGACAGCCTCGCGCCGCCAAGCGGGCGCCTCCCGCCCGGCCGTTTC
GCGCCCGCAGGCAAGCGGCGCCGTGGGATCGTGGAAAGTCGGCGATCGGGCGAATCACC
GGAAATGGGGCATCGGCACCGTCGTCAGCGTCCGCGGCGGCGGCGACGACCAAGAGCTC
GACATCGCCTTCCCGAGCCCGATCGGCATTAAACGGTTGCTTGCCAAATTTGCGCCGATTG
AGAAAGTGGGTAGCGGCGACTACAAGGACGATGACGATAAGGATTATAAAGACGATGACGA
TAAGGACTACAAAGACGATGACGATAAATAGGGATCC

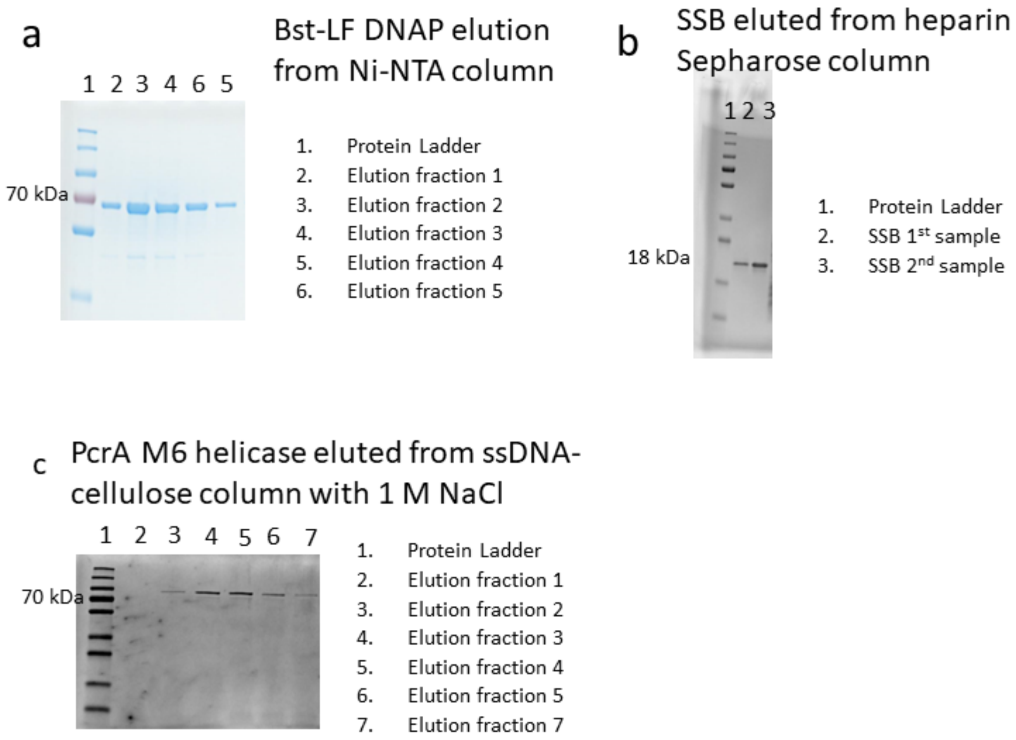

Supplementary Fig. 1 Qualitative SDS-PAGE gel analysis was used to shows purified proteins used in SHARP. a Bst-LF DNAP is purified using Ni-NTA column. b SSB is purified by ammonium sulfate precipitation followed by heparin Sepharose column. c PcrA M6 is purified using Ni-NTA column followed by ssDNA-cellulose column.

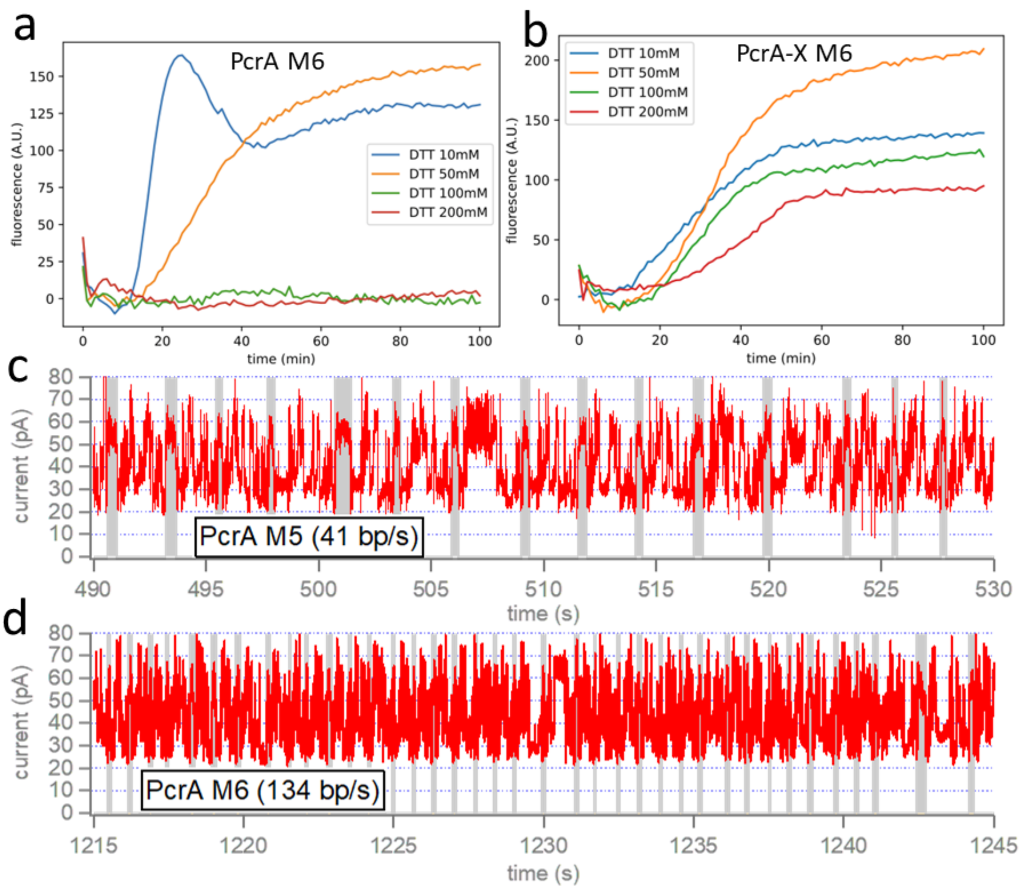

Supplementary Fig. 2 Effects of DTT on SHARP and SPRNT traces. a Increased DTT concentration (>50 mM) inhibits SHARP, as expected because DTT breaks disulfide bonds essential for the superhelicase activity of PcrA M6 in the absence of externally applied crosslinker. b Increased DDT concentration had low effect on SHARP activities of cysteine-crosslinked PcrA-X M6 with BM(PEG)2 c, d Longer section of nanopore signals for PcrA M5/M6 are used to calculate the average helicase speed.

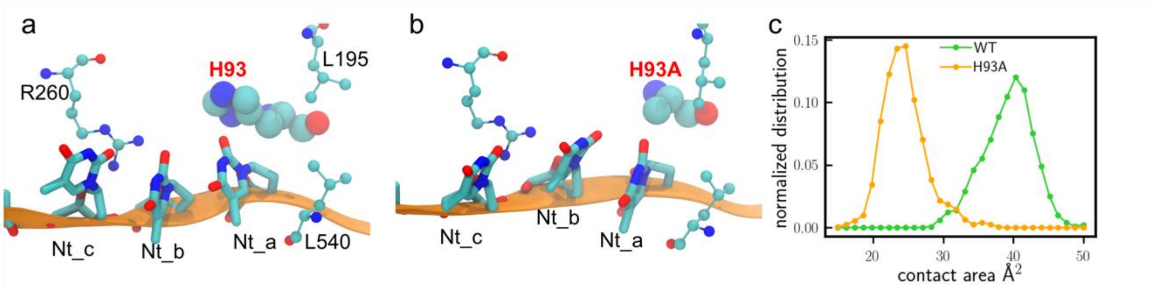

Supplementary Fig. 3 Interactions between H93/H93A and ssDNA at the pre-translocation state in MD. a and b. ssDNA-PcrA interactions at the initial (pre-translocation) state for PcrA (a) and H93A c Distributions for the contact areas at the initial state (green curve for Nt_a - H93; orange curve for Nt_a - H93A)
