# Supplementary material for: Engineered helicase replaces thermocycler in DNA amplification while retaining desired PCR characteristics
Source: Nat Commun. 2022 Oct 23;13:6312. doi: 10.1038/s41467-022-34076-0 (PMC9588791; doi:10.1038/s41467-022-34076-0)
Supplement: Supplementary file 3 — Reporting Summary [file 41467_2022_34076_MOESM3_ESM.pdf]

Corresponding author(s): Taekjip Ha

Last updated by author(s): Sep 24, 2022

Reporting Summary

Nature Portfolio wishes to improve the reproducibility of the work that we publish. This form provides structure for consistency and transparency in reporting. For further information on Nature Portfolio policies, see our Editorial Policies and the Editorial Policy Checklist.

Statistics

For all statistical analyses, confirm that the following items are present in the figure legend, table legend, main text, or Methods section.

n/a	Confirmed	
☐	☒	The exact sample size (*n*) for each experimental group/condition, given as a discrete number and unit of measurement
☐	☒	A statement on whether measurements were taken from distinct samples or whether the same sample was measured repeatedly
☐	☒	The statistical test(s) used AND whether they are one- or two-sided<br>*Only common tests should be described solely by name; describe more complex techniques in the Methods section.*
☒	☐	A description of all covariates tested
☒	☐	A description of any assumptions or corrections, such as tests of normality and adjustment for multiple comparisons
☐	☒	A full description of the statistical parameters including central tendency (e.g. means) or other basic estimates (e.g. regression coefficient) AND variation (e.g. standard deviation) or associated estimates of uncertainty (e.g. confidence intervals)
☐	☒	For null hypothesis testing, the test statistic (e.g. *F*, *t*, *r*) with confidence intervals, effect sizes, degrees of freedom and *P* value noted<br>*Give P values as exact values whenever suitable.*
☒	☐	For Bayesian analysis, information on the choice of priors and Markov chain Monte Carlo settings
☒	☐	For hierarchical and complex designs, identification of the appropriate level for tests and full reporting of outcomes
☒	☐	Estimates of effect sizes (e.g. Cohen's *d*, Pearson's *r*), indicating how they were calculated

Our web collection on statistics for biologists contains articles on many of the points above.

Software and code

Policy information about availability of computer code

Data collection	BioRad CFX Manager version 3.1. was used to collect DNA amplification data in real time.<br>Amersham Imager 600 from GE Healthcare and its built-in software were used to image gels.<br>Cary Eclipse fluorometer with Kinetics Software Version: 1.1(132) collected helicase activity assays.<br>Thermofisher NanoDrop 2000 was used to measure concentrations of DNA templates and purified proteins.<br>Axon Axopatch 200B Microelectrode Amplifier and National Instruments DAQ card and LabVIEW 2018 built-in voltage-recording function (DAQ Assistant) were used to collect the nanopore data.<br>GPU-accelerated NAMD (version 2.13) was used to perform the MD simulations. All the algorithms related to MD (e.g. SHAKE) are implemented and available in the NAMD software.
Data analysis	Amplification time was determined using the built-in function in CFX Manager version 3.1.<br>IgorPRO 6.0 was used to fit amplification times assuming exponential model and obtain doubling time for all templates.<br>Image J was used to visually inspect gel images and determine the presence of amplicon bands.<br>Primer-template pairs and plasmid sequences were analyzed and constructed using SnapGene Viewer versions 5 and 6.<br>PcrA helicase structure was analyzed and plotted using PyMol verion 2.2.<br>VMD version 1.9.4 was used to analyze MD simulation results and render related molecular images.

For manuscripts utilizing custom algorithms or software that are central to the research but not yet described in published literature, software must be made available to editors and reviewers. We strongly encourage code deposition in a community repository (e.g. GitHub). See the Nature Portfolio guidelines for submitting code & software for further information.

Data

Policy information about availability of data

All manuscripts must include a data availability statement. This statement should provide the following information, where applicable:

- Accession codes, unique identifiers, or web links for publicly available datasets
- A description of any restrictions on data availability
- For clinical datasets or third party data, please ensure that the statement adheres to our policy

Source data are provided with this manuscript. Gel images, qSHARP, fluorescence, nanopore, and sequencing data generated in this study are provided in the Source Data file. Plasmid and protein sequence data for the engineered helicase are provided in the Supplementary Information.

Field-specific reporting

Please select the one below that is the best fit for your research. If you are not sure, read the appropriate sections before making your selection.

☒ Life sciences ☐ Behavioural & social sciences ☐ Ecological, evolutionary & environmental sciences

For a reference copy of the document with all sections, see nature.com/documents/nr-reporting-summary-flat.pdf

Life sciences study design

All studies must disclose on these points even when the disclosure is negative.

Sample size	We tested nearly 1300 reactions to optimize SHARP. Based on work by M. Vincent, Y. Xu and H. Kong, "Helicase-dependent isothermal DNA amplification," in EMBO reports, vol. 5, p. 795–800, 8 2004., we designed initial SHARP protocol. The initial reaction showed poor performance. Then, we selected one component to optimize (i.e Helicase, SSB, DNAP, DTT, primer concentration and type...) and tested up to 20 reactions where the selected component concentration was varied. The condition giving the fastest amplification time and the correct gel band was considered optimal. For this optimal condition, we varied another component in 20 reactions and selected the new optimum. The process was iteratively repeated until SHARP performance matched the reference PCR. Optimal SHARP components are summarized in Table 1. For the optimal condition in Table 1, we tested different primer-template sets.<br>In the nanopore assay, for both PcrA M5 and M6 we analyzed 1e4 steps to determine the average unwinding speed.<br>For the quantification of genome editing data in Fig. 4, we used two separate cell cultures for each case. The total of 16 biologically independant cell cultures.
Data exclusions	No data were excluded from the analysis.
Replication	We replicated SHARP result with more than 40 conditions. We showed several different primer-template sets (155 bp, 200 bp, 1.4 kbp, 3 kbp, 6 kbp, (CAG)47, gDNA) at various template concentrations. All attempts to replicate SHARP were successful.
Randomization	The experiments were not randomized. The randomization is not relevant for the iterative optimization process of SHARP, because the outcome from the previous reaction determines the next reaction to be tested.
Blinding	Blinding was implemented only for the reaction involving gDNA from human cells. M.G. premixed SHARP components according to Table 2 and provided premixed RxnMix, EnzMix, and protocol to R.Z.. R.Z. carried out amplification of gDNA with custom primers and premixed RxnMix and EnzMix without knowing composition of RxnMix and EnzMix.<br>For all other reactions blinding was not relevant, because in the optimization process the investigator has to find what known condition gives the best amplification result in order to design the next reaction.

Reporting for specific materials, systems and methods

We require information from authors about some types of materials, experimental systems and methods used in many studies. Here, indicate whether each material, system or method listed is relevant to your study. If you are not sure if a list item applies to your research, read the appropriate section before selecting a response.

Materials & experimental systems

n/a	Involved in the study	
☒	☐	Antibodies
☐	☒	Eukaryotic cell lines
☒	☐	Palaeontology and archaeology
☒	☐	Animals and other organisms
☒	☐	Human research participants
☒	☐	Clinical data
☒	☐	Dual use research of concern

Methods

n/a	Involved in the study	
☒	☐	ChIP-seq
☒	☐	Flow cytometry
☒	☐	MRI-based neuroimaging

Eukaryotic cell lines

Policy information about cell lines

Cell line source(s)	HEK293T from ATCC, catalog # CRL-3216
Authentication	None of the cell lines used were authenticated
Mycoplasma contamination	None of the cell lines used were authenticated
Commonly misidentified lines (See ICLAC register)	None
