# Supplementary figures and images for: Engineered helicase replaces thermocycler in DNA amplification while retaining desired PCR characteristics
Source: Nat Commun. 2022 Oct 23;13:6312. doi: 10.1038/s41467-022-34076-0 (PMC9588791; doi:10.1038/s41467-022-34076-0)

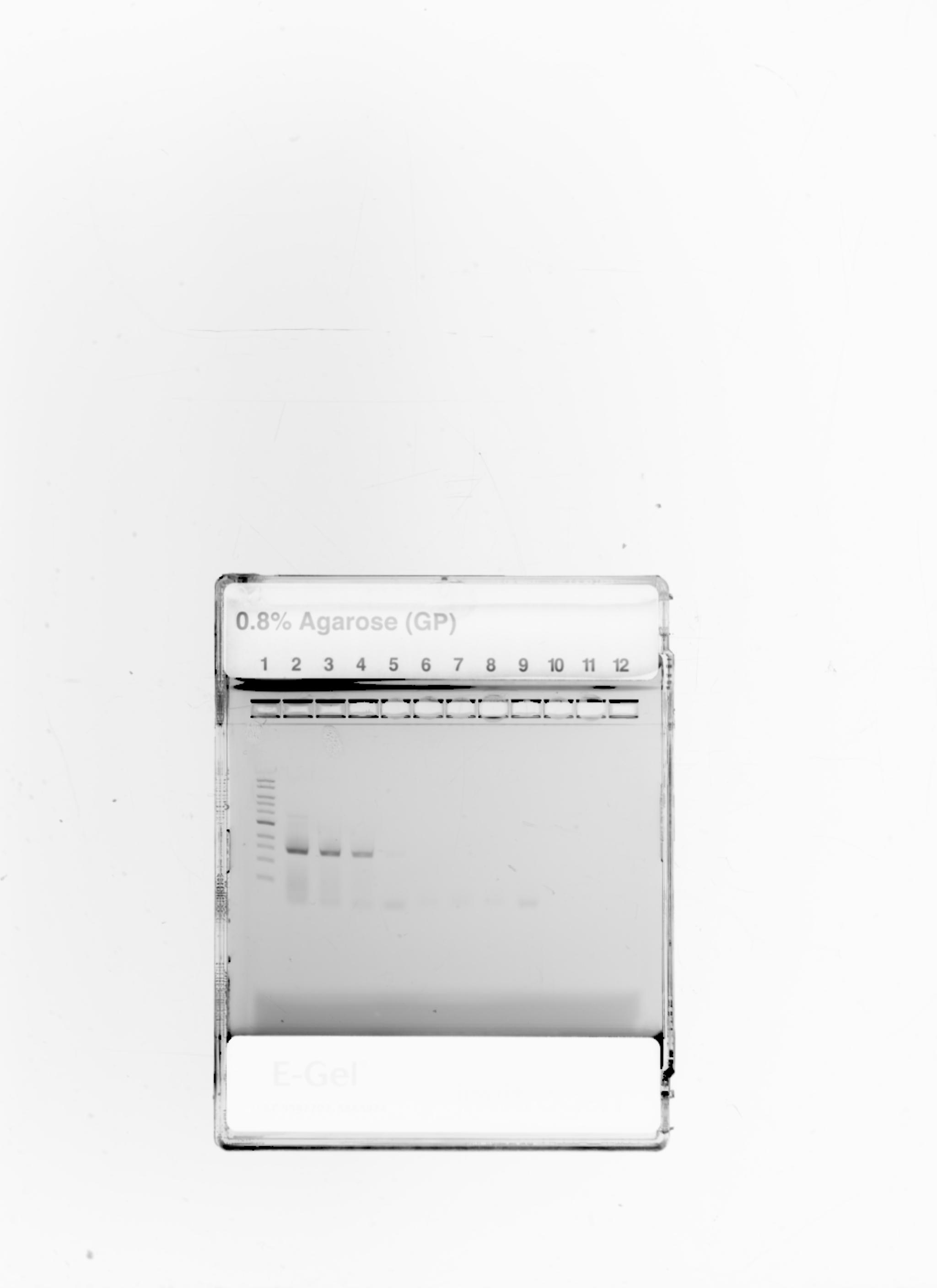

Supplement: Supplementary file 4 — Source Data [file 41467_2022_34076_MOESM4_ESM.zip › 5_SourceDataFiles/Fig2/Fig2b_Mo_PCR4 2020.10.15_08.14.52_Fl-UV.tif]

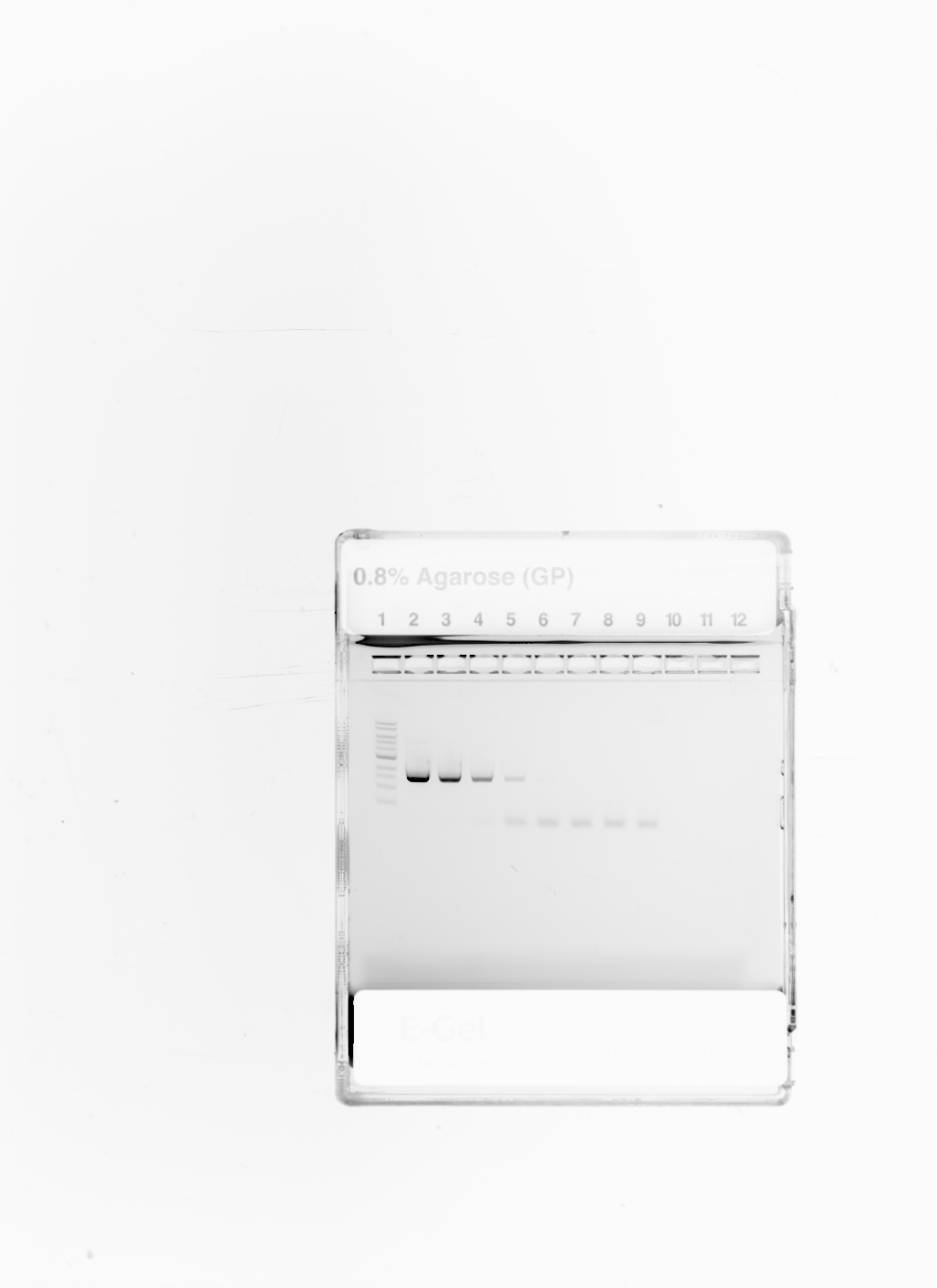

Supplement: Supplementary file 4 — Source Data [file 41467_2022_34076_MOESM4_ESM.zip › 5_SourceDataFiles/Fig2/Fig2d_Mo_SHARP_dLim4 2020.10.13_10.16.56_Fl-UV.tif]

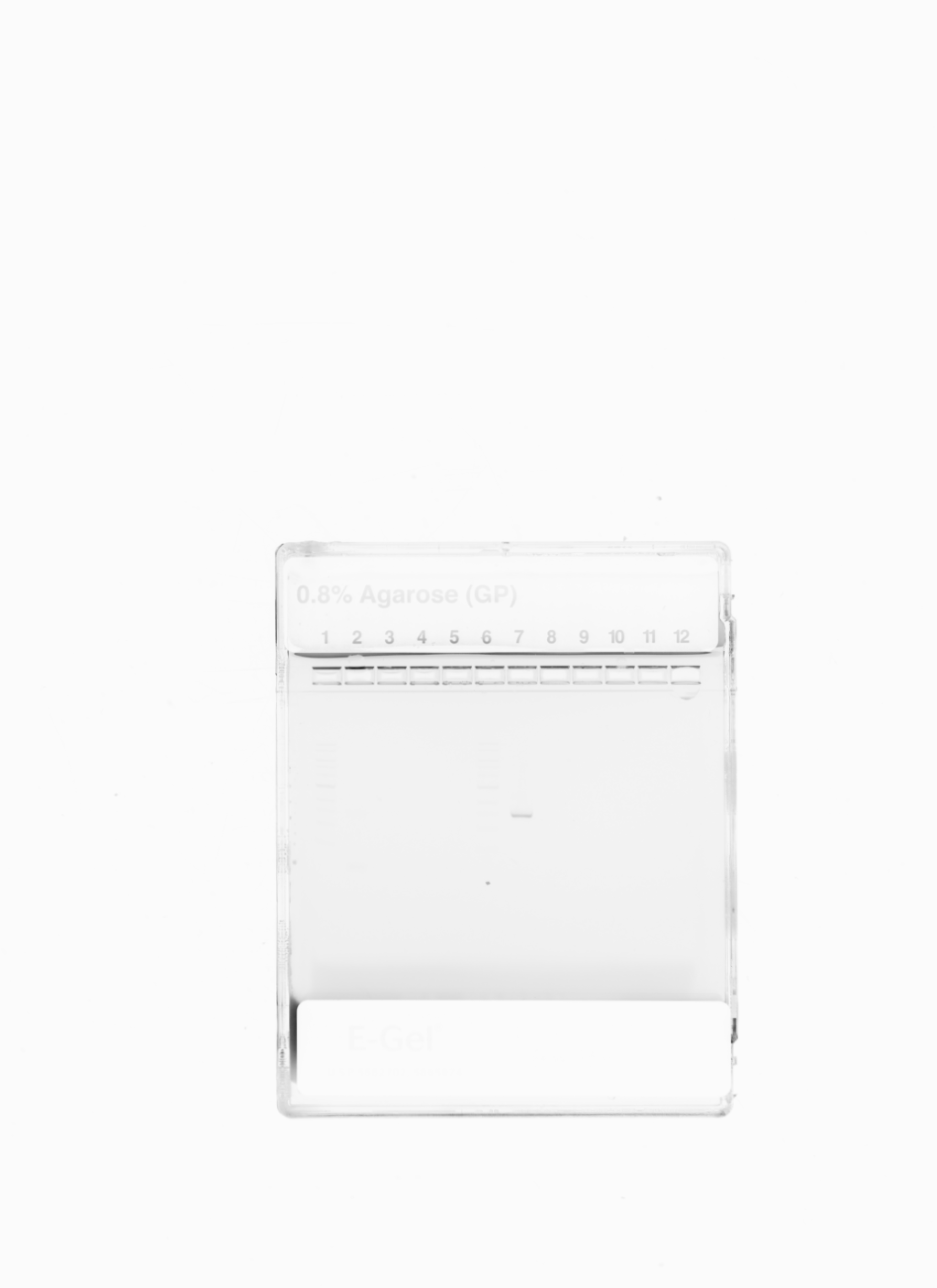

Supplement: Supplementary file 4 — Source Data [file 41467_2022_34076_MOESM4_ESM.zip › 5_SourceDataFiles/Fig2/Fig2f_MG_4 2021.04.06_11.46.12_Fl-UV.tif]

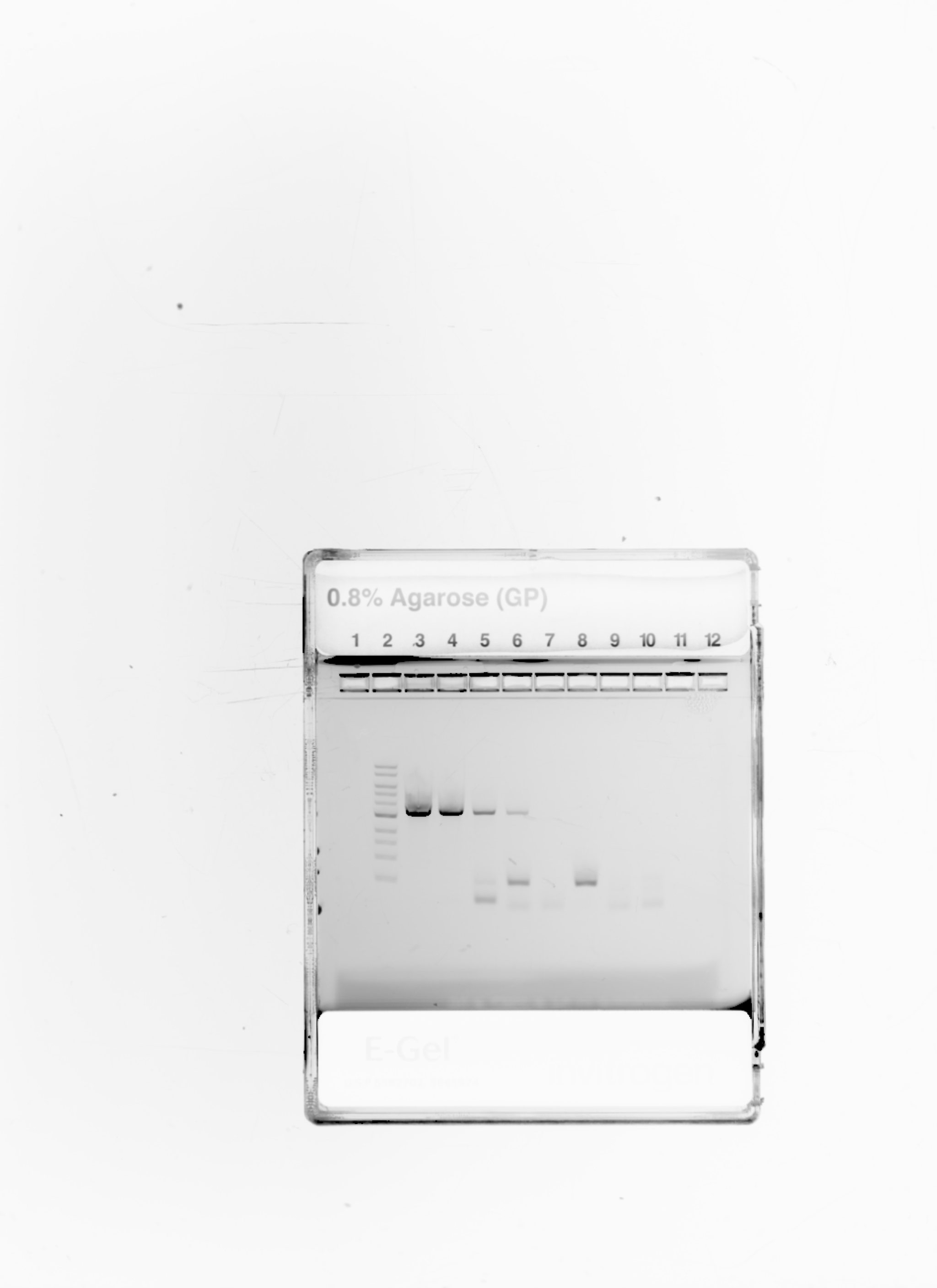

Supplement: Supplementary file 4 — Source Data [file 41467_2022_34076_MOESM4_ESM.zip › 5_SourceDataFiles/Fig3/Fig3a_Mo_2.3kbpSHARP3 2020.10.19_10.24.56_Fl-UV.tif]

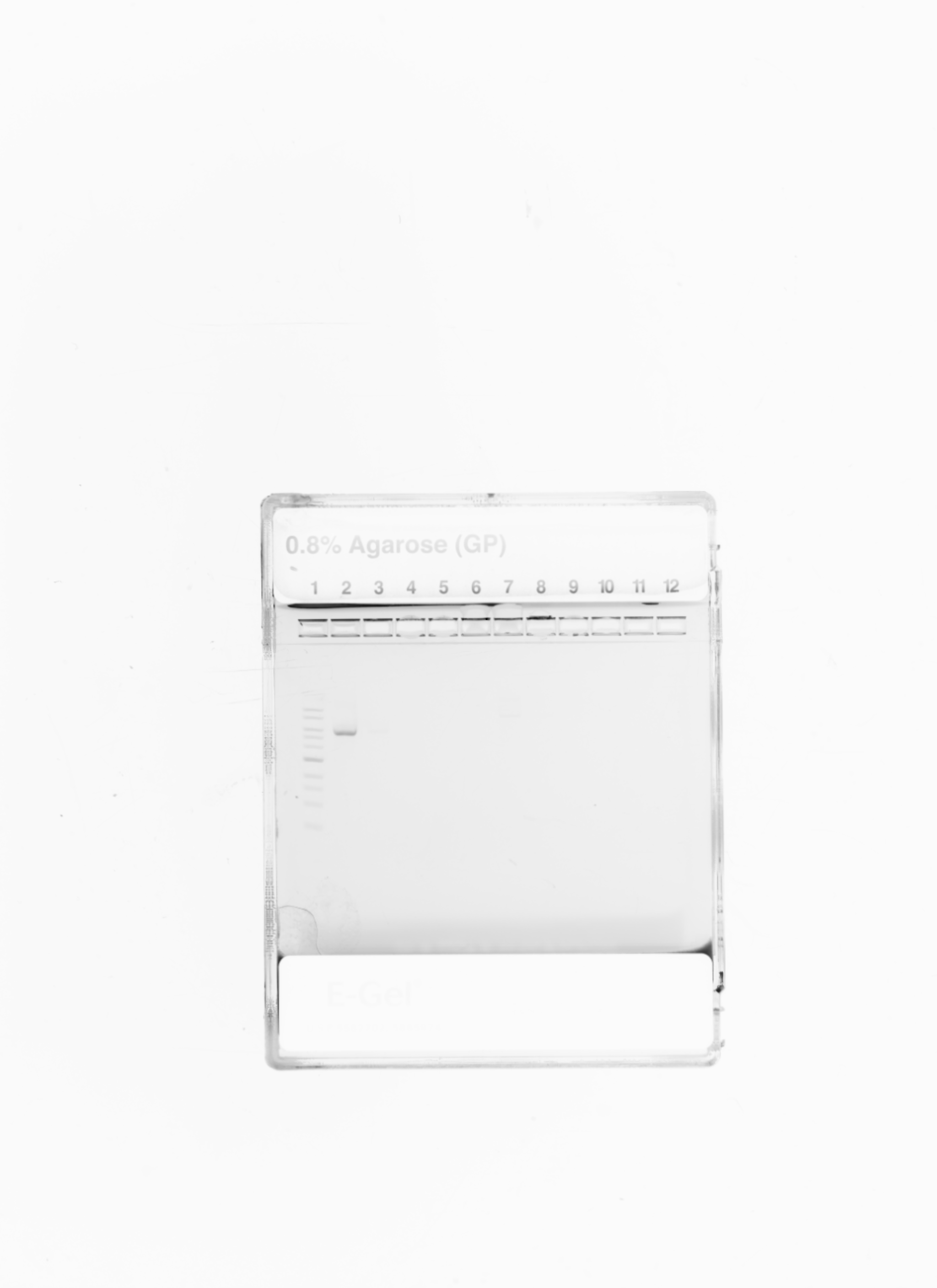

Supplement: Supplementary file 4 — Source Data [file 41467_2022_34076_MOESM4_ESM.zip › 5_SourceDataFiles/Fig3/Fig3b_Mo_ChinDNA_Lambda2 2020.10.29_15.42.00_Fl-UV.tif]

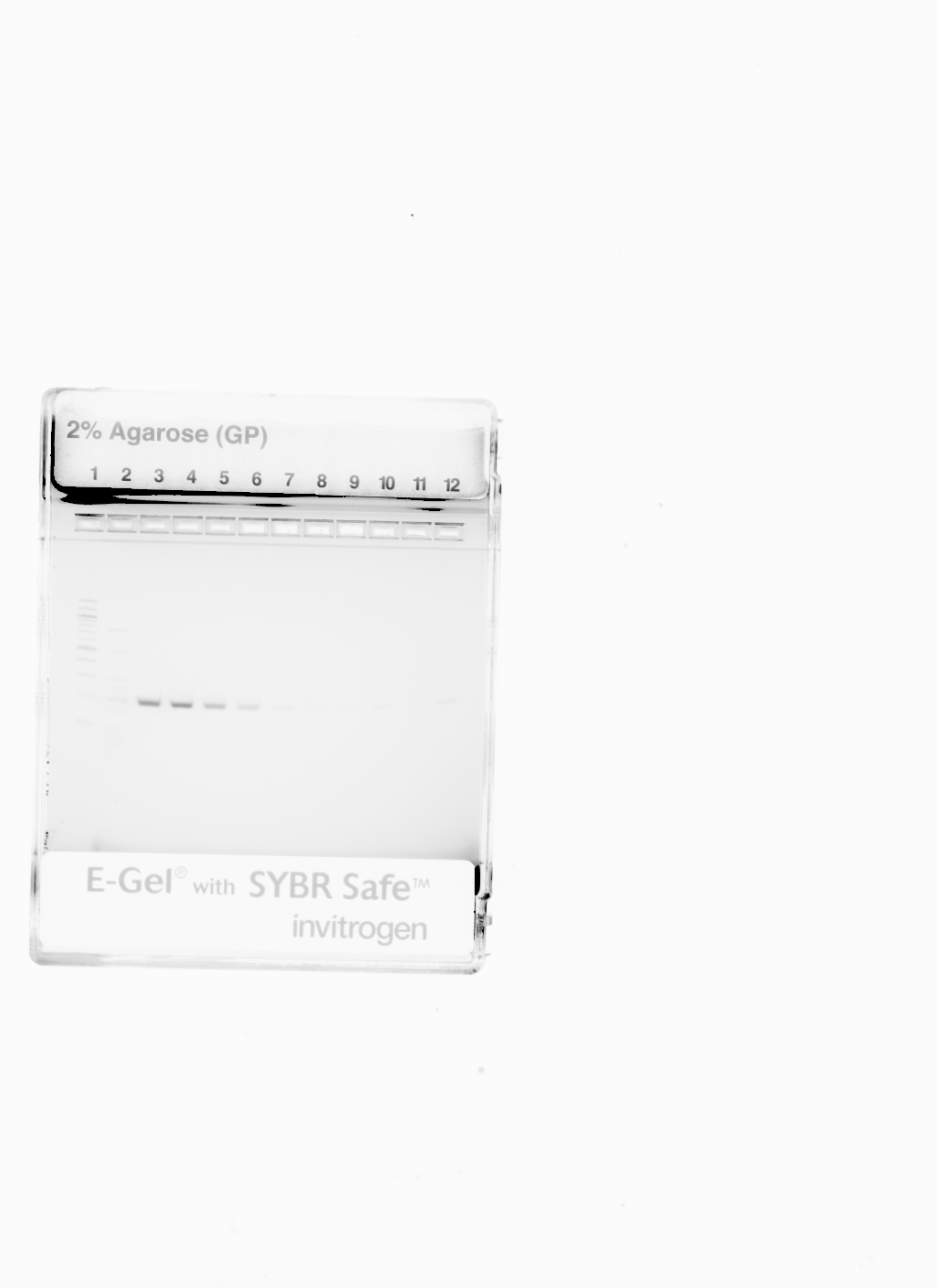

Supplement: Supplementary file 4 — Source Data [file 41467_2022_34076_MOESM4_ESM.zip › 5_SourceDataFiles/Fig3/Fig3c_MG_200bp_lambda2 2021.03.22_17.52.53_Fl-Blue.tif]

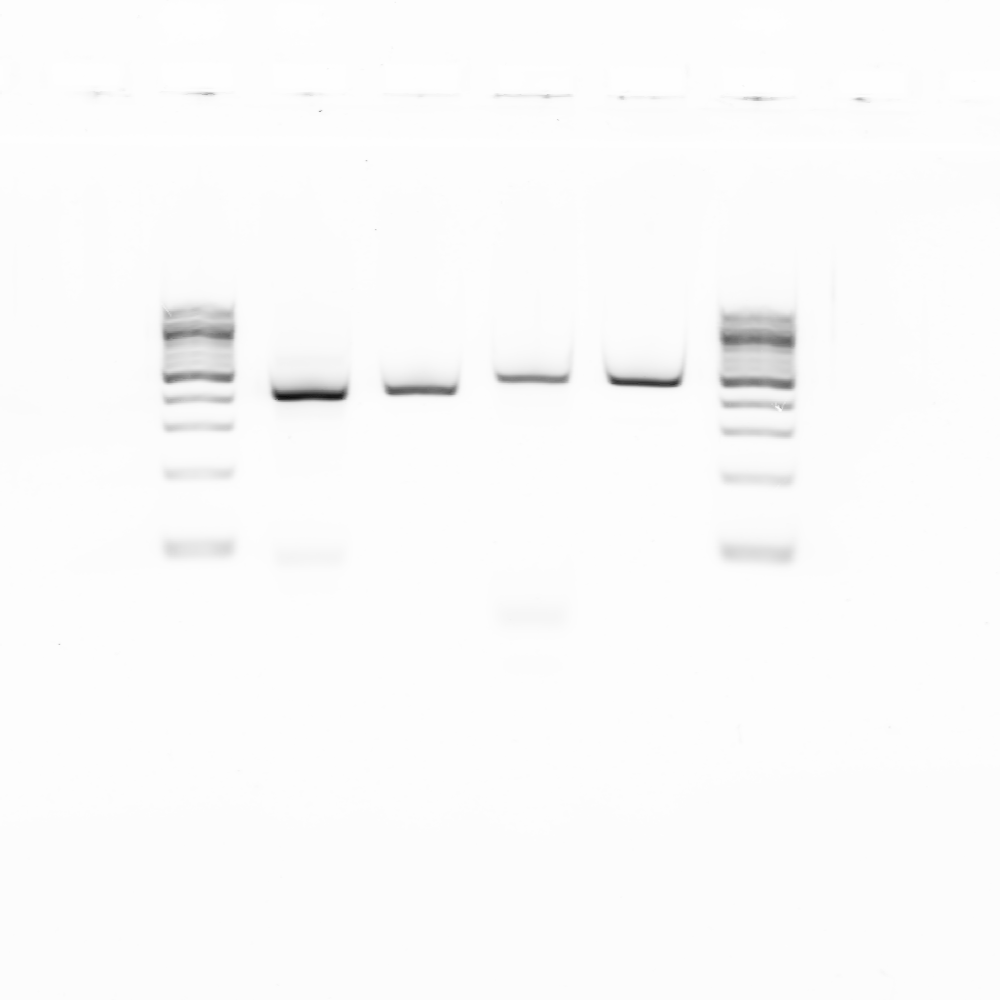

Supplement: Supplementary file 4 — Source Data [file 41467_2022_34076_MOESM4_ESM.zip › 5_SourceDataFiles/Fig4/Fig4b_rep1.tif]

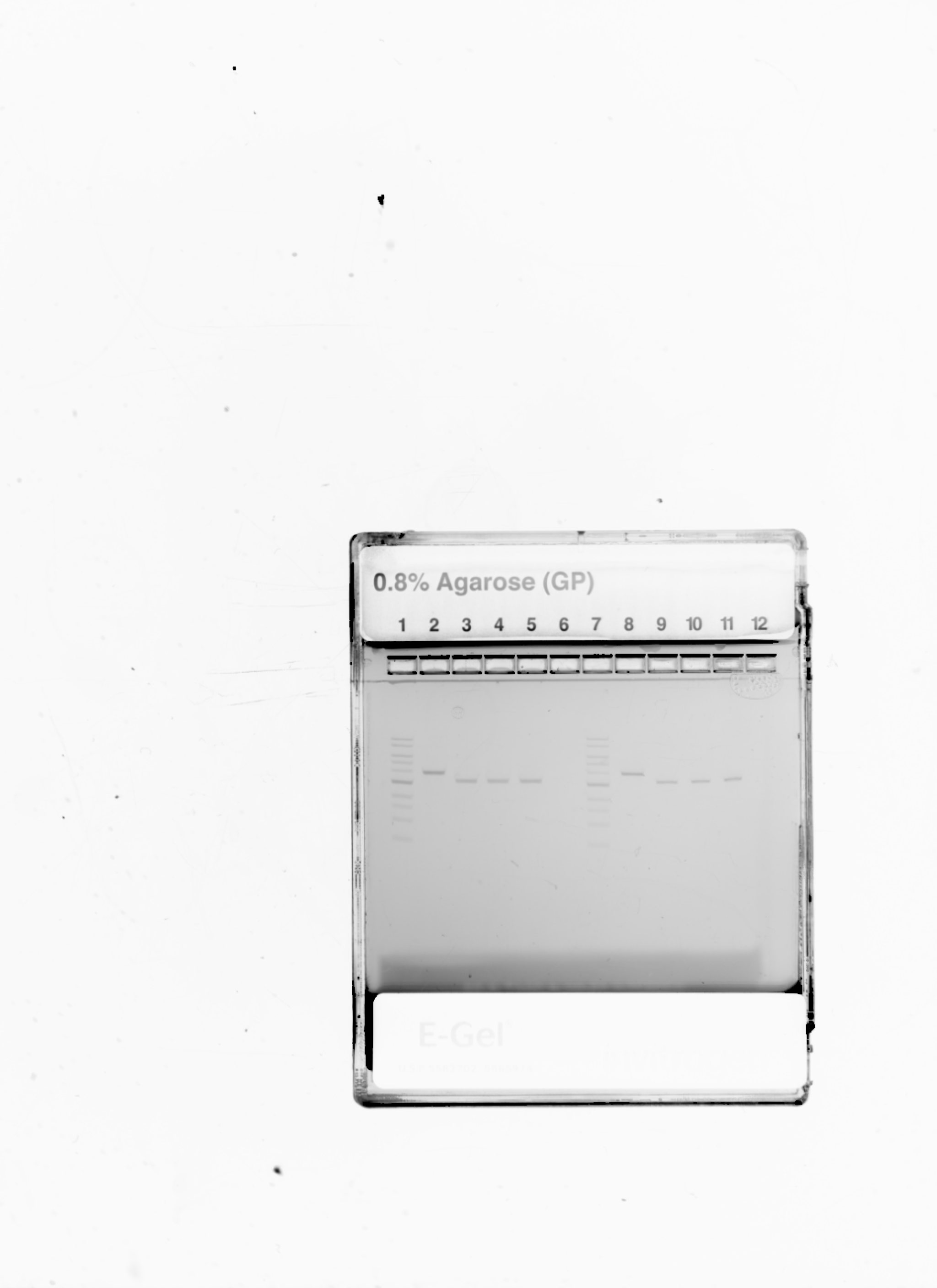

Supplement: Supplementary file 4 — Source Data [file 41467_2022_34076_MOESM4_ESM.zip › 5_SourceDataFiles/Fig5/Fig5a_MG_3.2cells6 2020.12.18_15.57.57_Fl-UV.tif]

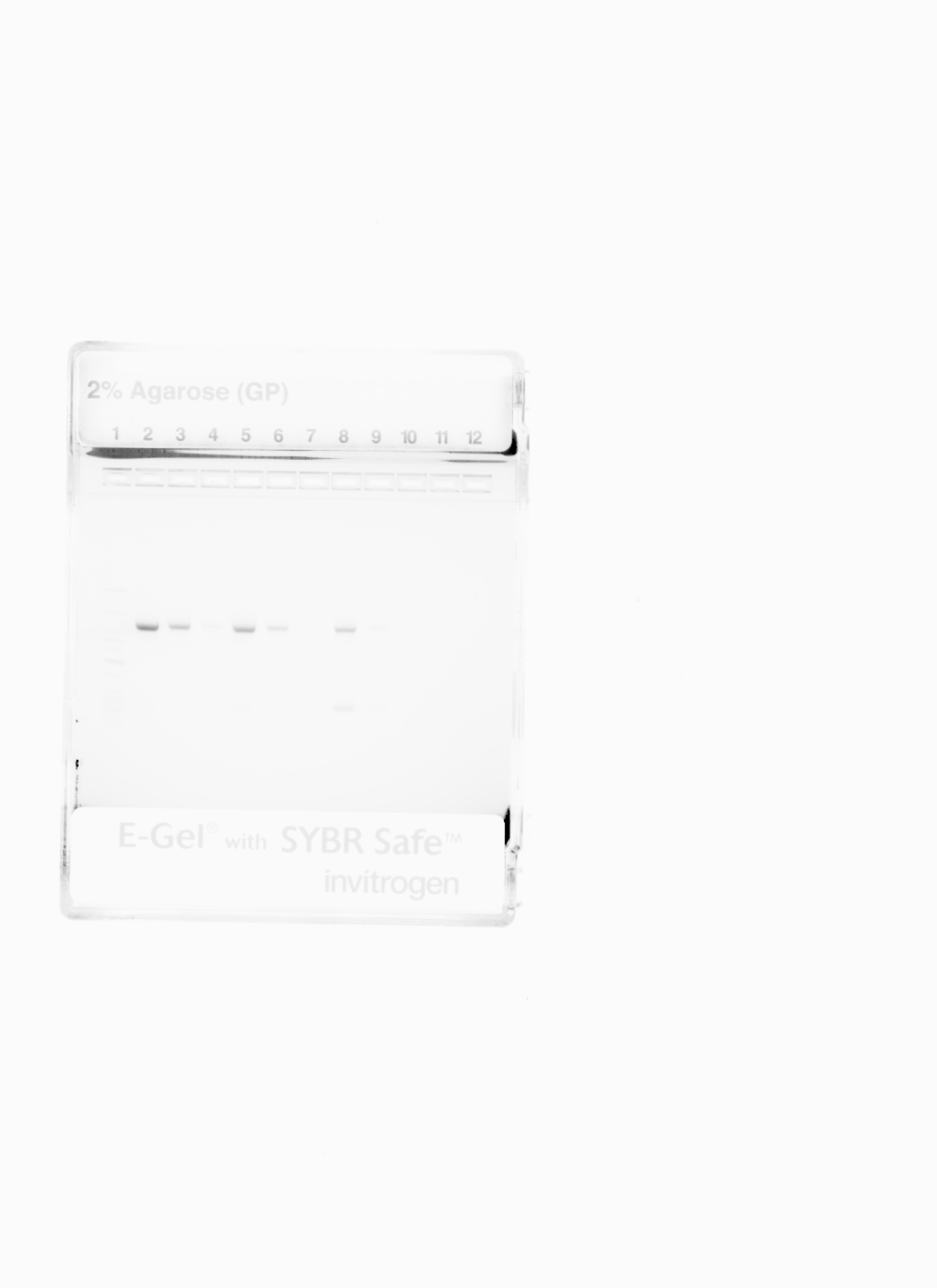

Supplement: Supplementary file 4 — Source Data [file 41467_2022_34076_MOESM4_ESM.zip › 5_SourceDataFiles/Fig5/Fig5b_MG_CAG4 2021.02.27_18.56.03_Fl-Blue.tif]

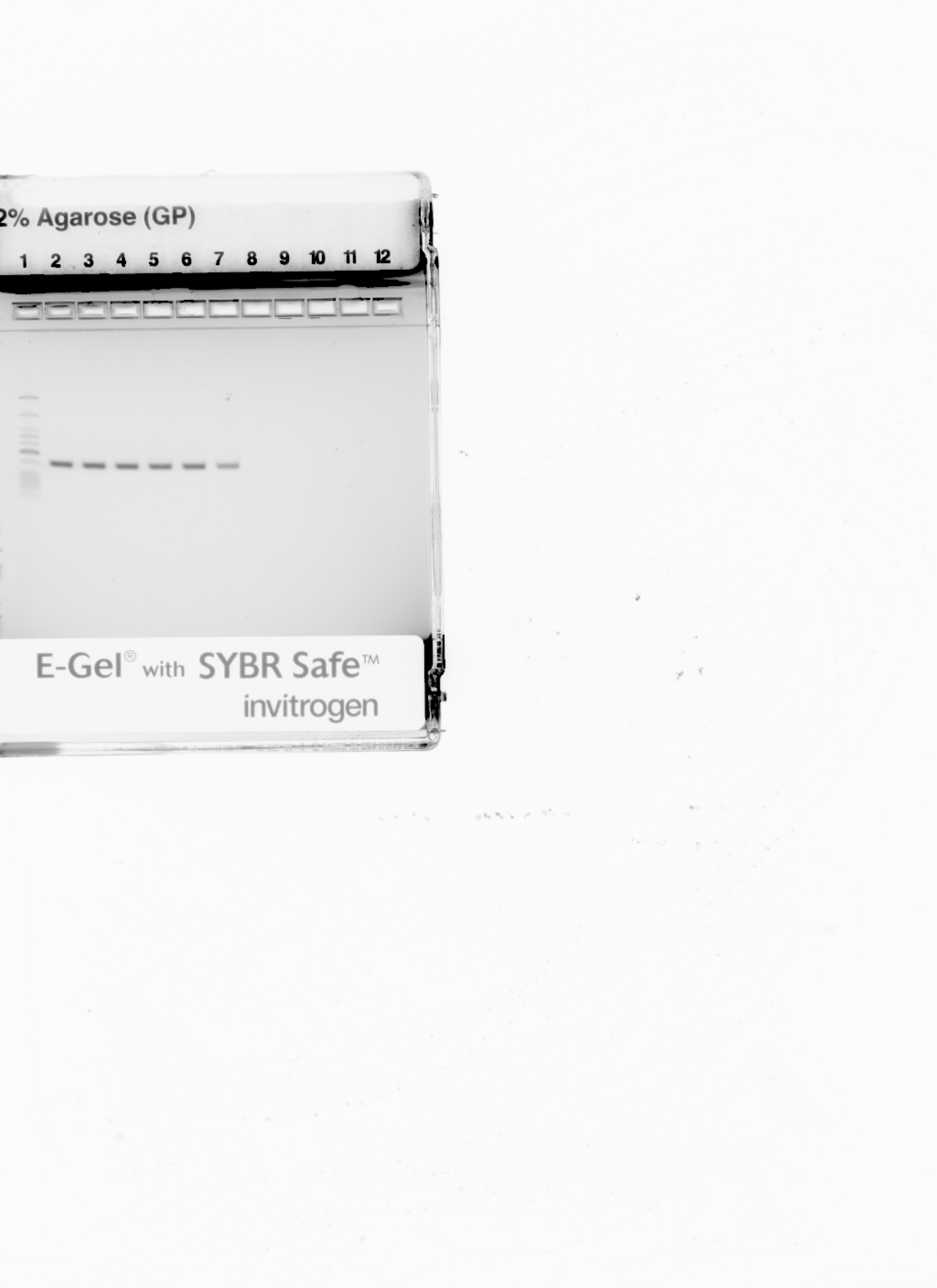

Supplement: Supplementary file 4 — Source Data [file 41467_2022_34076_MOESM4_ESM.zip › 5_SourceDataFiles/Fig5/Fig5d_MG6 2021.04.13_12.29.49_Fl-Blue.tif]

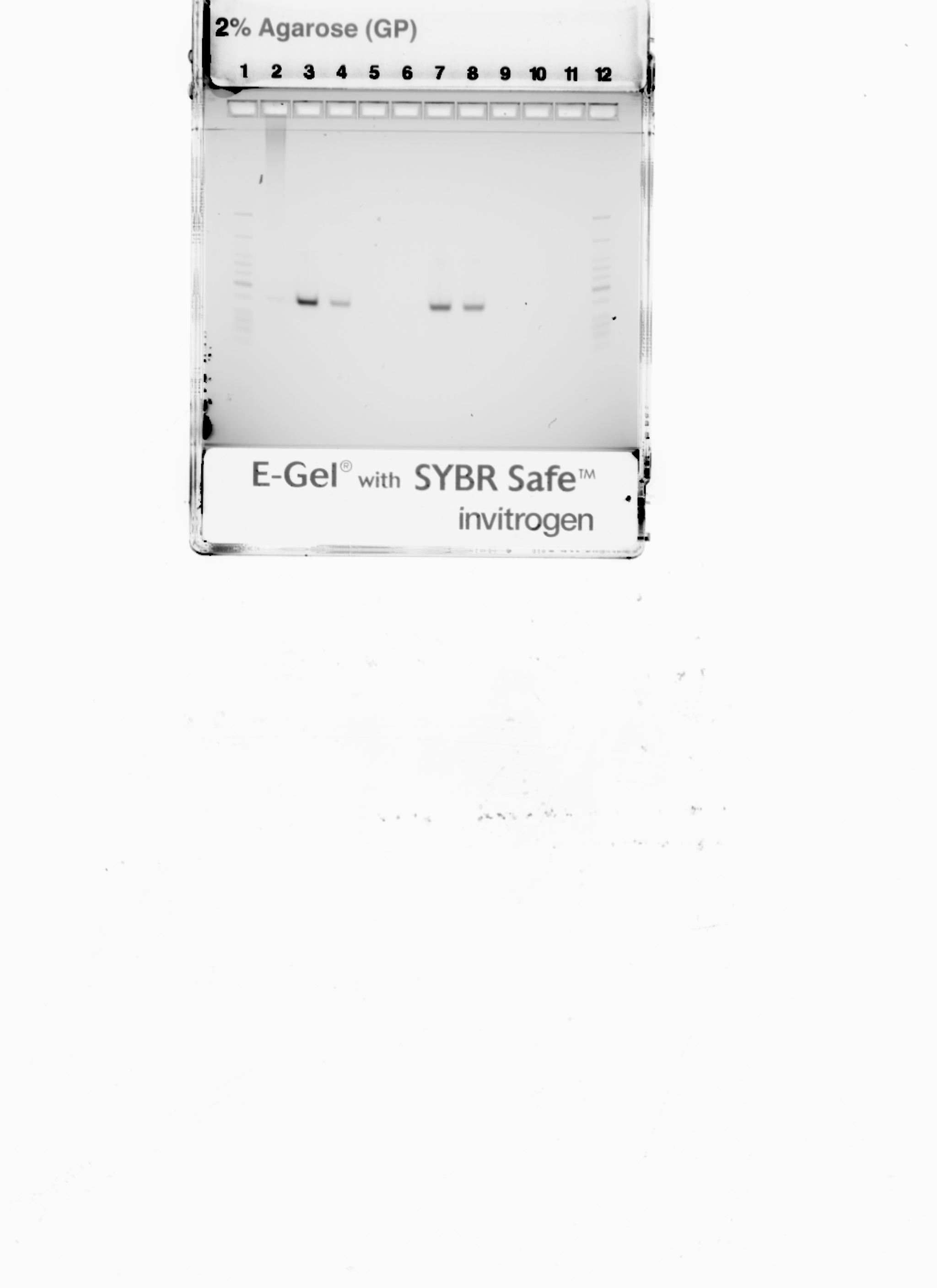

Supplement: Supplementary file 4 — Source Data [file 41467_2022_34076_MOESM4_ESM.zip › 5_SourceDataFiles/Fig5/Fig5e_MG_Bsu3 2021.06.16_11.16.40_Fl-Blue.tif]
